# Supplementary material for: Leptin Reduces Plin5 m6A Methylation through FTO to Regulate Lipolysis in Piglets
Source: Int J Mol Sci. 2021 Sep 30;22(19):10610. doi: 10.3390/ijms221910610 (PMC8508756; doi:10.3390/ijms221910610)
Supplement: Supplementary file 1 [file ijms-22-10610-s001.zip › ijms-1364130-supplementary.pdf]

Table S1 Primer sequences

Gene	Primer sequences（5’-3’）	Accession number
Leptin R-F	TATCGTGCAGTCGCTCAGTG	NM_001024587.1
Leptin R-R	TCAGGCTCCAAAAGAAGAGGG	
Plin5-F	CTGGTCAGGACCACATGCAC	NM_001123135.1
Plin5-R	ACGAGATCATTCACGGTGGC	
Plin2-F	TTGCTGCTGGTCGATTTCTT	NM_214200.2
Plin2-R	CACTTCCGGTCACTGCTTCT	
Plin1-F	TGGTGGCGTCTGTATGCAAT	NM_001038638.1
Plin1-R	CAGCACCGAGGACTTTGTCT	
PPARγ-F	AACATTTCACAAGAGGTGACCA	NM_214379.1
PPARγ-R	GATCTCGTGGACGCCATACT	
HSL-F	CTAGCAAACATGGCATCGGC	NM_214315.3
HSL-R	CCAGCATAGGCACTGACACA	
ATGL-F	TGTTCCCCAAAGAGACGACG	NM_001098605.1
ATGL-R	CGTTGGCCACTAGGGAGGA	
LPL-F	CGTGCTCAGATGCCCTACAA	NM_214286.1
LPL-R	AGACTCCACGTGCTGTTCCT	
ACC1-F	ACCCCAGGAGGATGTGCTAT	NM_001114269.1
ACC1-R	AGCTGGCTAGTGGAGGTGT	
Fasn-F	TGGGCATGGTGAACTGTCTC	NM_001099930.1
Fasn-R	GCGTGGTTGTTGGAAAGGTC	
PGC-1α-F	ATGTGCAACCAGGACTCTGT	NM_213963.2
PGC-1α -R	GCGTCTCTGTGAGAACTGCT	
CPT1a-F	TTCAGTTCACGGTCACTCCG	NM_001129805.1
CPT1a-R	TGGATCCCAGGAGAATCGGT	
TFAM-F	TGCTTTGTCTACGGGTGCAA	NM_001130211.1
TFAM-R	GCAAAACTGAACGGAGAGCG	
Ndufb8-F	CCCAGGACCCTATCCCAAGA	NM_001144842.2
Ndufb8-R	CCAGTGTATCGGTTCACCCC	
Sdhb-F	ACATCAACGGAGGCAACACT	NM_001104953.1
Sdhb-R	ACAGCCCATCCAGTTTCTCG	
Uqcrc2-F	CTCCTGTAAGGCGGTTGTGA	XM_003124555.5
Uqcrc2-R	ACTGGATGCAAGACGAAGCA	
COX4i1-F	GGTGGAGTCCCCTCTCGAT	XM_021093705.1
COX4i1-R	GGATGGGGCCGTACACATAG	

Atp5a1-F	TCGTGGTGTTCGTCTGACTG	NM_001185142.1
Atp5a1-R	TTTTCCCAACAGGGCTTGGT	
ABHD5-F	GGATGCCTGTGAGAGGTCTG	NM_001012407.1
ABHD5-R	GGTGTGACGTCGATGTAGGG	
Mettl3-F	ACACTGCTTGGTTGGTGTCA	XM_003128580.5
Mettl3-R	AATCTTTCGAGTGCCAGGGG	
Mettl14-F	GTGGTTCTGGGGAGGGATTG	XM_003129231.6
Mettl14-R	TCCACCTCCCCGATCAGATT	
FTO-F	GGCATGGGGTCATCCTTTGA	NM_001112692.1
FTO-R	CCAGGGGTCTCTATGTCCCA	
Ythdf2-F	AACAAGGGTCCTGTGGCAAA	XM_005665152.3
Ythdf2-R	GCTGTGTCTGTTGCCCTACT	
UCP3-F	TCACCTTCAGGACACGTTCG	NM_214049.1
UCP3-R	AGGCATCCATCCTAGTGGGT	
Casp3-F	CCGGAATGGCATGTCGATCT	NM_214131.1
Casp3-R	AGTCCAATTCTGTGCCTCGG	
Casp9-F	CAGTGGTGCTGGGGTCTAAG	XM_013998997.2
Casp9-R	GGCCTTGGCAGTCAGGTT	
Bax-F	GCCCTTTTGCTTCAGGGTTTC	XM_003127290.5
Bax-R	CAATGCGCTTGAGACACTCG	
Bcl2-F	ATCAAGTGTTCCGCGTGACT	XM_021099593.1
Bcl2-R	GGCCCATACAGCTCCACAAA	
ACSL2-F	TCAGTCCTTCCTCCGATGAT	NM_001167629.2
ACSL2-R	AGCTTCCGCTGTTTTTGCTG	
DGAT2-F	TCTACTTCACTTGGCTGGCG	NM_001160080.1
DGAT2-R	GCTTGGAGTAGGGCATGAGC	
β-Actin-F	AACGGCTCCGGCATGTGCAA	NM_007393
β-Actin-R	CTTCTGACCCATGCCCACCA	
